# Data Scaling: Implications for Climate Action and Governance in the UK

**DOI:** 10.1007/s00267-024-01991-5

**Published:** 2024-05-29

**Authors:** Andrew Sudmant

**Affiliations:** 1 Edinburgh Climate Change Institute, University of Edinburgh, Edinburgh, UK; 2 Sustainability Research Institute, Faculty of Environment, University of Leeds, Leeds, UK

**Keywords:** Climate change, Climate action, Polycentric governance, Governance, Modifiable areal unit problem, Ecological fallacy, Place-based climate action

## Abstract

Local actors have growing prominence in climate governance but key capacities and powers remain with national policymakers. Coordination between national and local climate action is therefore of increasing importance. Underappreciated in existing academic and policy literature, coordination between actors at different scales can be affected not only by politics and institutional arrangements, but also by methods of data analysis. Exploring two datasets of GHG emissions by local area in England—one of consumption-based emissions and the other of territorial emissions—this paper shows the potential for a data scaling problem known as the modifiable areal unit problem and its possible consequences for the efficacy and equity implications of climate action. While this analysis is conceptual and does not identify specific instances of the modifiable areal unit problem or its consequences, it calls attention to methods of data analysis as possible contributors to climate governance challenges. Among other areas, future analysis is needed to explore how data scaling and other aspects of data processing and analysis may affect our understanding of non-state actors’ contribution to climate action.

## Research highlights

- Data scaling could affect the efficacy and equity of climate action in the UK.
- Enabling local actors can help to overcome the effects of data scaling.
- Relationships between methods of data analysis and governance need more attention.
- Research is needed on data scaling and the climate actions of non-state actors is needed.

## Introduction

The growing number and diversity of actors playing a role in the fight against climate change is widely recognised as a positive development for the climate movement. A greater diversity of actors is suggested to lead to more dynamic and innovative approaches to governance (Jordan et al. 2015). Devolution and depoliticization of responsibility for action has the potential to support more effective decision-making (Romero-Lankao et al. 2018). The bringing of stakeholders and citizens closer to the decision making processes is seen to increase the democratic legitimacy of climate actions (Cattino and Reckien 2021). And higher levels of engagement facilitated by polycentric governance can help to build a social mandate for action (Howarth et al. 2020). Evaluating the climate action commitments of actors including local governments, schools, universities, communities and individuals is a growing area of research (Grafakos et al. 2020; Messori et al. 2020).

A growing number and diversity of actors can also, however, increase the challenge of coordinating action, potentially leading to duplication of effort, mixed signals to households and businesses, and conflicting climate actions (Biermann, 2014; Bulkeley, 2010; Li and Shapiro 2020). Understanding how the growing involvement of non-state and sub-national actors is affecting climate action requires detailed policy and place specific analysis, and the continued developed of theory (Bulkeley, 2010; Bulkeley and Betsill 2005; Gouldson et al. 2016; Howarth et al. 2020; Willis et al. 2022; Creutzig et al 2024). Missing from existing analysis, however, is consideration of the way methods of data analysis, independent from institutional arrangements and mobilisations of power, can affect actor’s understanding of the climate challenge.

Using datasets of GHG emissions at the local level in the UK, this analysis provides conceptual insight into the way methods of data analysis can affect climate action by demonstrating the potential for the modifiable areal unit problem. The modifiable areal unit problem has been highlighted as affecting our understanding of the impact of climate change on agriculture (Cai, 2021; Deschênes and Greenstone, 2007) and in the context of adaptation and climate resilience (Hutton et al. 2011). To the knowledge of the author, however, research has not considered how the modifiable areal unit problem applies to climate change mitigation.

Analysis focuses on the UK due to the unique conditions of climate governance that have developed over the last decade in the country. At the local level, efforts to develop and nurture local, and so-called ‘place-based’, climate actions have surged over recent years in the UK. Since 2019, 314 (of 408) local authorities have declared Climate Emergencies, 16 local authorities have developed Climate Commissions (place-based non-government bodies responsible for supporting local climate action), and 210 local authorities have established net-zero targets for decarbonisation before the 2050 date set out in national legislation.

In Section “The Modifiable Areal Unit Problem and Climate Mitigation” we outline our approach, including how we model the modifiable-areal-unit problem and the way we explore how the modifiable-areal-unit problem might have consequences for the efficacy and equity implications of climate action. Following this, in Section “Methodology” we present the methodology. In Section “Results: The Effect of Data Scaling on Climate Action Priorities in England” we explore how the modifiable-areal-unit problem may lead to conflicting climate priorities between national and local actors in the UK. In Section “Discussion” we discuss what the possible implications of the modifiable-area-unit problem for the efficacy for climate action and for the distribution of the costs and benefits of climate action. In Section “Conclusion” we reflect on the implications of this research for climate policy and the governance of climate change.

## The Modifiable Areal Unit Problem and Climate Mitigation

The modifiable-areal-unit problem consists of two related issues. A scaling issue can occur when data is aggregated, for example, when moving from a local to a regional scale of analysis. At each stage of aggregation, characteristics that are unique to any local area can become harder to identify at higher level geographies. A zonation issue can emerge when data is aggregated into different configurations, for example the splitting of a region into electoral districts. In this analysis only the scale effect is considered. Hereafter we refer to the modifiable-area-unit problem as the ‘data scaling problem’.

**Fig. 1:**
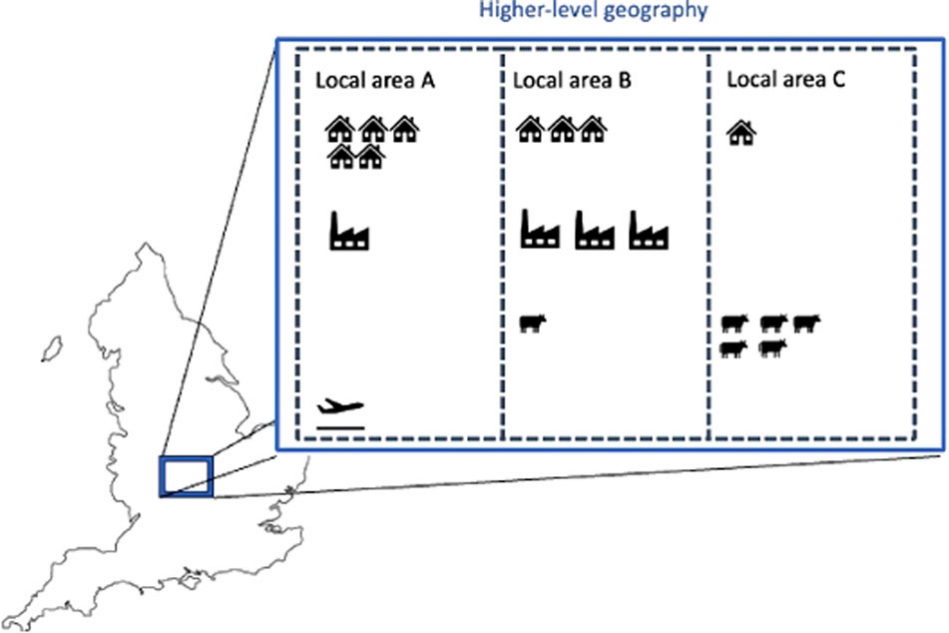
A simplified representation of the modifiable areal unit problem. Each icon represents a unit of emissions with different icons representing different sources of emissions in a region in England. The cow represents agriculture, the house represents the domestic sector, the airplane represents aviation, and the factory represents industry

Figure 1 provides an example of a scale effect emerging via the modifiable areal unit problem. For each of local areas A, B and C, different sources of emissions (represented by icons that each represent one unit of GHG emissions) are of relatively greater and lesser importance. Local area A, for example, has no emissions from agriculture (represented by the cow), while agriculture is the largest source of emissions for local area C. Climate action plans for each local area would therefore be likely to focus on different sources of emissions. Local area A, for example, would be more likely to focus on housing, while C would be more likely to focus on agriculture. A climate plan focusing on the emission *across* these local areas, however, would be likely to view housing as its highest priority – since it is the largest source of emissions - followed by agriculture.

Different priorities for climate action within local areas compared with across local areas could lead to consequence for the effectiveness of climate action. If the climate action plans focused on a single priority area, a plan developed across local areas would address 9 units of emissions from the housing sector (5 + 3 + 1), while climate plans in each area would focus on 13 units of emissions (5 + 3 + 5). Individual climate plans could therefore lead to more effective climate action by generating more opportunities for intervention.

Different priorities for action between the local and higher-level geographies could also have equity implications. If climate actions are costly for households and businesses, the climate plan developed at the higher-level geography could impose more costs on area A than B or C due to the housing sector being relatively larger in area A. Conversely, if climate actions generate net benefits—because there are subsidies from government or through energy savings or the co-benefits of climate action—area A could benefit over areas B and C.

This example, and the approach taken in this paper, offer a hugely simplified representation of the way climate action plans are developed and of the possible efficacy and equity implications of these plans. Climate action plans at all levels are developed in the context of wider policies and priorities, existing plans, and the plans of wider actors, rather than a narrow focus on the scale of GHG emissions (Gouldson et al. 2020; Grafakos et al. 2020, Balouktsi, 2019; Dale et al., 2019; Howarth and Parsons 2021). The efficacy and equity implications of plans depend on factors that include the measures implemented (Millward-Hopkins et al. 2017), the sector considered (Mayrhofer and Gupta 2016) the socio-economic context (Colenbrander et al. 2017) and the approach to implementation (Klenert et al. 2018).

Setting these considerations aside dramatically narrows the scope of conclusions that can be drawn from this analysis. In particular, analysis will not be able to identify specific instances of the modifiable areal unit problem or its consequences. Using real GHG data however, allows this analysis to investigate an underexplored question: how the methods applied during data analysis—even when the same data are used by different actors—may contribute to different perspectives on the climate challenge. Exploring this question expands our understanding of the factors affecting the governance of climate action.

## Methodology

Two datasets are used in this analysis. The first consists of 32,844 emissions footprints from Lower Layer Super Outputs Areas (LSOAs) covering 12 sources of emissions. LSOAs are a geographic designation that includes an average population of 1500 people. This dataset was developed by the Centre for Research into Energy Demand Solutions (CRED) and is available from their place-based carbon calculator (Malcolm et al. 2021). The second dataset consists of territorial emissions across 407 local authorities between 2005 and 2021 (BEIS 2024). Local authorities have an average population of approximately 165,000. Local authorities have responsibilities over planning and development, waste management, and key aspects of transport, making them key actors in climate action planning and delivery.

The dataset from BEIS contains territorial emissions, the accounting approach used by the UK at the national level and the accounting approach applied by most climate actors in the UK. The CRED dataset provides data to the LSOA level, providing insight into community-level mitigation planning. The CRED dataset is developed around consumption-based emissions footprints, an approach to emissions accounting that includes supply-chain emissions.

Each of these datasets have limitations. The Malcolm et al. (2021) consumption-based dataset uses national data for estimating the emissions associated with some forms of consumption and downscales GHG data from the national level on a population basis. These necessary but simplifying steps, reduce the differences in emissions between areas, obscuring potential scaling issues. The dataset from BEIS (2024) are only to a local authority level and are reported with a 2-year lag. These limitations notwithstanding, using two different datasets and accounting approaches help to provide a more robust analysis of the potential for a data scaling problem.

The following equations are used in this analysis. To calculate GHG average per capita emissions for a given community:

1 $${{\rm{GHG}}_{j}}=\sum{{\rm{GHG}}_{{\rm{i,j}}}}/\sum{\rm{p}}_{i}$$

Where GHG_j_ is average per capita GHG emissions in sector *j*, GHG_i,j_ is average GHG emissions of sector *j* in community *i*, and *p*_*i*_ is the population of community *i*. To sum across multiple communities as we move from a local to a regional or national geography i∈n sums across the set of local communities in the higher-level geography:

2 $${{\rm{GHG}}_{j,n}}=\mathop{\sum}\limits_{{\rm{i\in n}}}{{\rm{GHG}}_{{\rm{i,j}}}}/\mathop{\sum}\limits_{{\rm{i\in n}}}{\rm{p}}_{i}$$

To understand how the scaling of emissions could affect climate action, analysis compares the share of total emissions covered when climate action plans are developed at different scales using different geographic aggregations of data.

Priority areas for climate action are determined based on the relative scale of sources of emissions where a sector with larger emissions is identified as a higher priority than a sector with lower emissions. As previously highlighted, this approach provides a simplified representation of the way priorities are set for climate action. The purpose of applying this simplified approach is to establish in a clear and transparent way the potential for methods of data analysis to effect approaches to climate action.

Net-zero climate targets are generally understood to require all sources of emissions to achieve near zero emissions in the near future, either through emissions mitigation measures or through a combination of mitigation measures and carbon removals (Peters 2018). Climate actions plans at both the national and local level, however, frequently highlight specific sources of emissions in their near-term focus (cf Reckien et al. 2018). Consequently, the share of total emissions covered by a climate plan developed at a local level will not always be the same as the share covered by a plan developed at a higher geography.

Following the approach in Section “The Modifiable Areal Unit Problem and Climate Mitigation”, the potential efficacy implications of data scaling are explored through the share of emissions covered by different climate action plans, with a larger coverage of emissions assumed to be associated with a more effective climate action plan. In the example in Fig. 1, for example, more emissions are covered when the single largest sector at each local geography is focused on, compared with focusing on the largest source of emissions across all geographies.

Similar to the approach in Section “The Modifiable Areal Unit Problem and Climate Mitigation”, the potential equity implications of data scaling are considered by comparing the share of total emissions covered between local level (LSOA) climate action plans. Looking across local areas more variation in the share of emissions to be covered by climate actions is interpreted as greater potential for equity implications from climate action. For example, if in one scenario some local areas are required to target 100% of their emissions and others target 0% of their emissions, the approach here determines that this would lead to a more unequal distribution of costs and benefits from climate action than if all local areas were required to address the same share of their emissions.

## Results: The Effect of Data Scaling on Climate Action Priorities in England

**Fig. 2:**
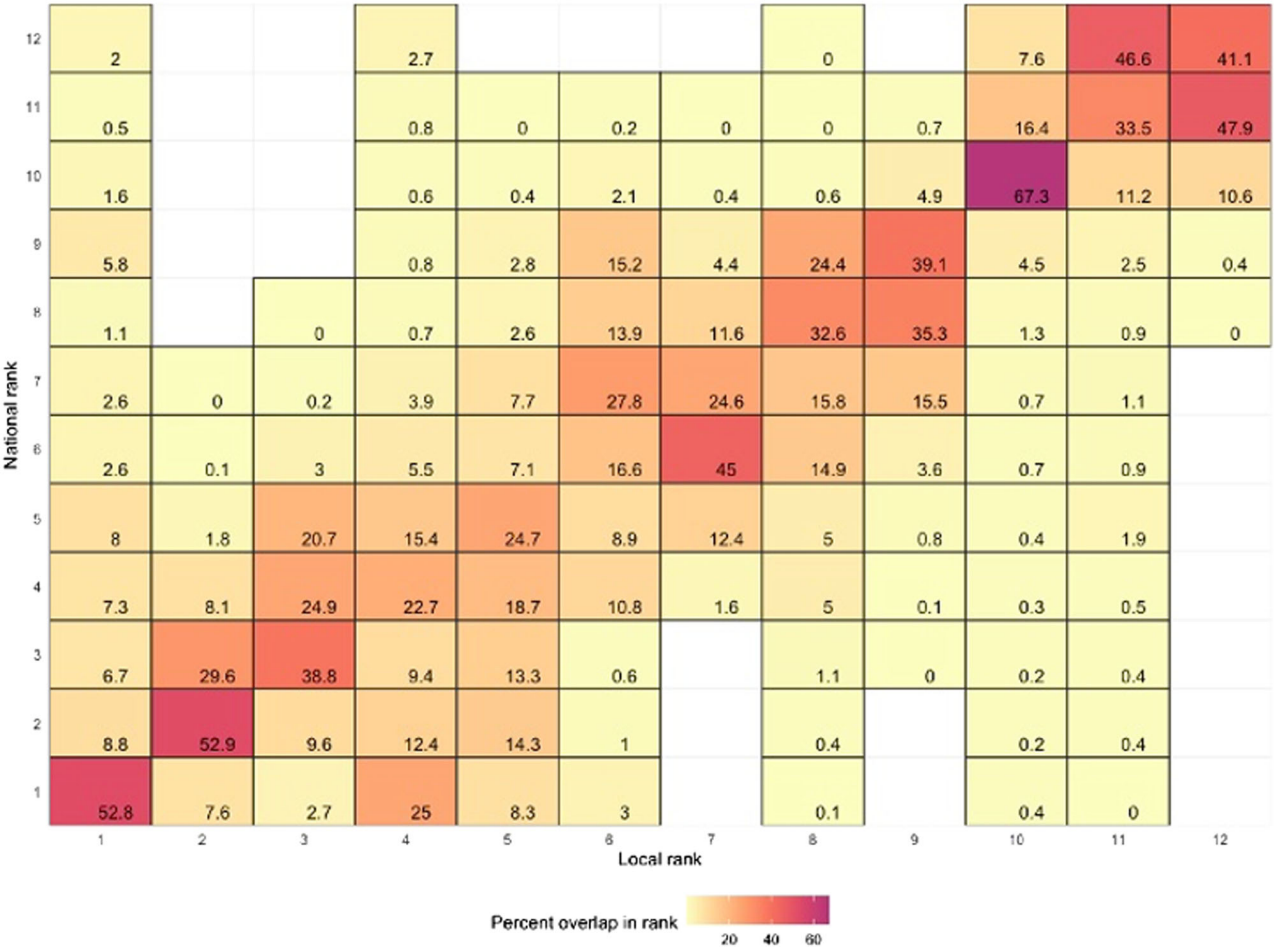
Rank of sources of emissions at the national versus LSOA level. Consumption-based emissions, 12 sources of emissions. Malcolm et al. (2021)

**Fig. 3:**
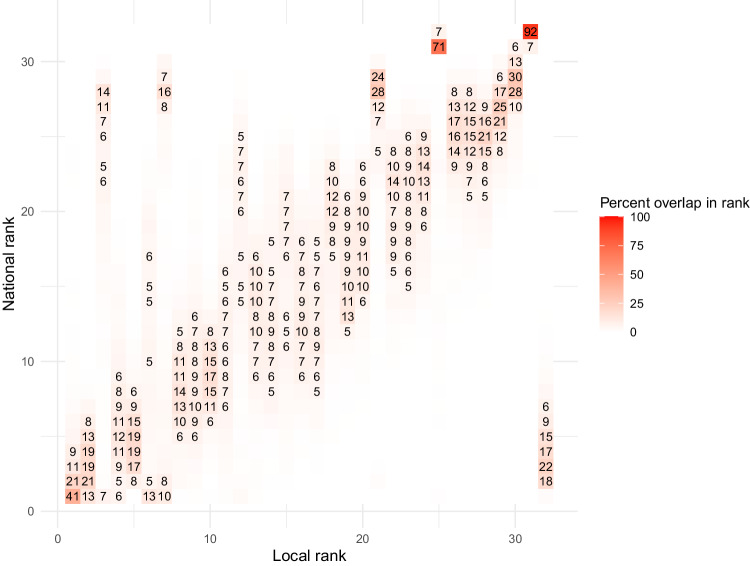
Rank of sources of emissions at the national versus local authority level. 33 sources of emissions, territorial emissions. BEIS 2024. For clarity values smaller than 5% have been removed

To understand how the scaling of data could lead to different approaches to climate action we can explore how sources of emissions are seen to be larger and smaller at different scales of analysis. Figures 2 and 3 compare the rank of emissions sources at the lowest and highest geographies. Figure 2 investigates a dataset with 32,844 LSOAs and 12 sources of emissions while the Fig. 3 investigates a dataset with 407 local authorities and 33 sources of emissions. A higher percentage (and darker cell) indicates that the national ranking of a sector is more frequently the same as the local ranking of a sector. For example, the cell in the bottom left of Fig. 2 shows that 53% of the time the sector with the largest volume of emissions is the same at the national level and the LSOA level.

These figures demonstrate that the ranking of emissions by sector is meaningfully affected by the geography used for analysis. In Fig. 2 the largest source of emissions at the LSOA level is only among the largest three sources of emissions of the national level 68.3% of the time (52.8 + 8.8 + 6.7%). Similarly, in Fig. 3 the largest source of emissions at the local authority level is only among the largest three sources of emissions of the national level 73.8% of the time (40.4 + 22.2 + 11.2%). This suggests that if climate action at the national level prioritised the three largest sources of emissions, for approximately 3 in 10 local areas the largest source of emissions would not be addressed.

Figures 2, 3 shows how a national ranking of emissions by sector will be different from a local ranking. The degree to which this affects climate action, however, will depend on the size of sources of emissions and the number of sources of emissions included in a climate action plan.

**Fig. 4:**
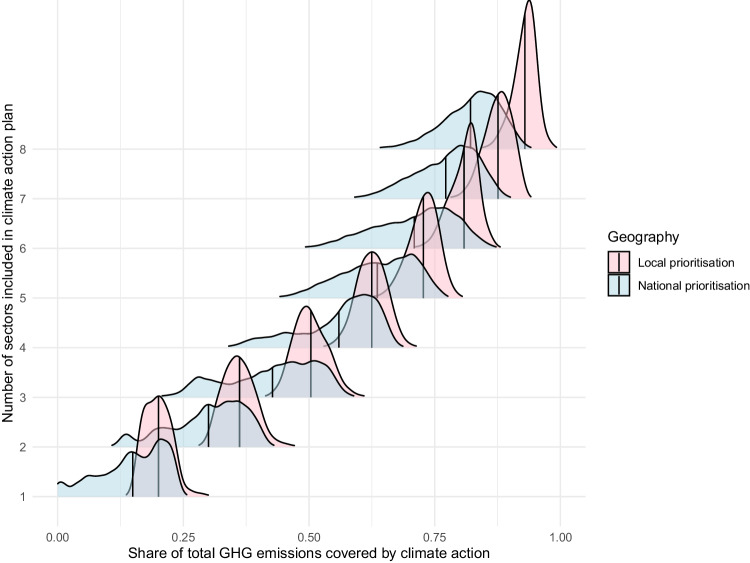
The density of LSOAs by the share of their emissions covered when local or national prioritisation guides action. The solid line shows the average. CRED data on consumption-based emissions. Local areas are LSOAs

**Fig. 5:**
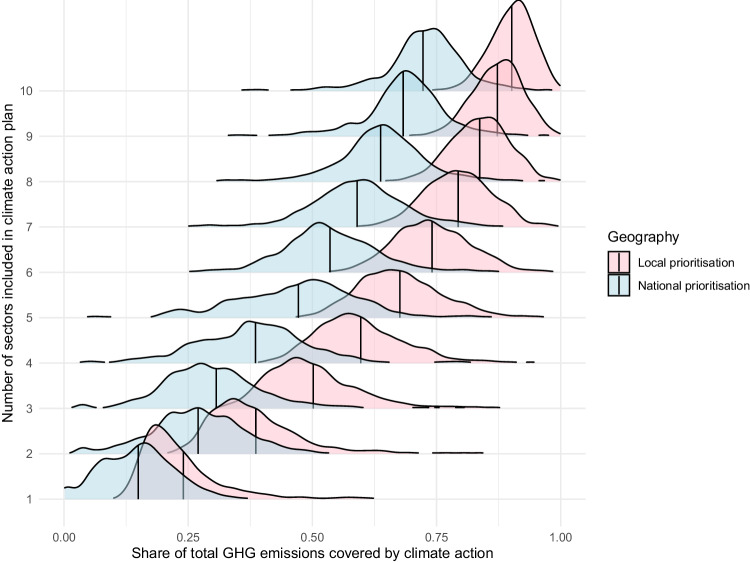
The density of LSOAs by the share of their emissions covered when local or national prioritisation guides action. The solid line shows the average. BEIS data on territorial-based emissions, 2005–2021. Local areas are local authorities

Figures 4, 5 compare the share of emissions covered by climate action plans derived from national (teal) or local (pink) prioritisation along the x-axis. Under national prioritisation (teal), all local areas address emissions from the same sources of emissions, with the set of sources of emissions that are prioritised determined by the ranking of sources of emissions by size at the national level. Under local prioritisation the largest sources of emissions specific to each local area are prioritised. The solid vertical lines show the average share of emissions covered by each approach. Along the y-axis are the number of sources of emissions included in a climate action plan.

Two insights can be drawn from Figs. 4, 5. First, while climate action plans developed from the sources of emissions largest in each local area cover a larger proportion of GHG emissions, the difference (identifiable by comparing the distance between the solid vertical blue and pink average lines in each distribution) diminishes as the number of sectors included increases. If three sources of emissions are considered, focusing on the priorities specific to each LSOA increases the coverage of a climate action plan by 28–35%. However, when half of emissions sources are covered by action, a coverage more similar to what is seen in typical climate action plans in the UK and internationally (c.f Colenbrander et al. 2019; Creutzig 2015; Millward-Hopkins et al. 2017; Williamson et al. 2020), the difference diminishes to 12–16%. For a typical local area, climate plans developed using national sources of emissions will therefore cover similar volumes of emissions as climate actions plans developed using local sources of emissions in cases where a large share of emissions sources are considered.

Second, Figs. 4, 5 show that share of total emissions covered by a climate action plan varies more between local areas when national prioritisation is used in the place of local prioritisation. This can be seen in the long tail of the blue (national) distributions in Figs. 4 and 5. Although the different in emissions coverage diminishes as more sectors are considered, for a subset of local areas the difference between national and local prioritisation stays quite large. Considering the case where half of all sources of emissions are considered, the LSOA in the 10^th^ percentile for coverage of its emissions would be addressing 62–76% of its emissions if national priorities were applied versus 72–84% if local priorities determined the sources of emissions to be addressed. For the local areas in the 10th percentile moving to local priorities would therefore increase the coverage of climate action plans by 20–25%.

Locally specific prioritisation therefore significantly improves the coverage of climate action for a subset of LSOAs. Identifying these communities may be important to improve the efficacy of climate action if covering a greater volume of emissions leads to more opportunities for climate action. Identifying these communities may also be important from an equity standpoint: If climate actions are costly, these places may be addressing less than their fair share of emissions. If climate action carries benefits, on the other hand, either indirectly via cleaner air or job opportunities, or directly via investment and government subsidies, these places may be receiving less than their fair share.

To explore which places may be most affected by this problem Table 1 shows the correlation between the English index of multiple deprivation and difference in coverage of emissions between national and local climate action plans. The English Index of Multiple Deprivation is a composite index that includes factors relating to income, employment, education, health, crime, barriers to housing and services, and living environment.

**Table 1:** Pearson’s Correlation Coefficient for the difference between the coverage of local and national climate action plans and LSOA level social indicators

Socio-economic indicator	Correlation with the difference between local and national prioritisation for the Malcolm (2021) dataset	Correlation with the difference between local and national prioritisation for the BEIS (2024) dataset
Multiple index of deprivation	0.08	0.11

Table 1 suggests that socio-economic status may be a predictor of the extent to which the national prioritisation of actions aligns with a local prioritisation. A higher index of multiple deprivation indicates more socioeconomic challenges. The positive correlation suggests that national and local climate action plans become more different as levels of deprivation increase. These results are statistically significant at the 1% level. These results do not provide any insight into a causative relationship.

## Discussion

### Data Scaling and the Efficacy and Equity Implications of Climate Action

Priorities for climate action at the national level almost inevitably differ from priorities for climate action in local areas. In part these differences reflect the divisions of powers and capacities between different levels of government. Political and ideological positions, past and existing approaches to climate action, and socio-economic context, among other factors, also affect what climate actions are prioritised (Bulkeley and Betsill 2005; Gouldson et al. 2014; Howarth and Parsons 2021). Here we show that differences in the prioritisation of climate actions between local and national actors could also be affected by methods of data analysis. When 3 or fewer sources of emissions are prioritised for action, climate action plans developed at the local level could cover 35% more emissions than climate action plans developed at the national level as a consequence of data scaling. To the extent climate action plans are more effective when they are targeted towards larger sources of emissions, these results show that data scaling could in theory impact the effectiveness of climate action.

Prioritisation in this analysis was determined only by assessing the scale of emissions from different sources, setting aside other factors that influence priority setting. While further work is needed to understand how other factors might reduce or increase the effects of data scaling, we would cautiously suggest these factors could increase the effects of scaling. Socioeconomic context, for example, would likely increase variation between local areas, making it harder for any set of climate actions at the national level to align with the priorities of local areas.

The potential for impact on the equity of climate actions may be more significant than the effect on the efficacy of climate action. Recent analyses suggest that the transition to net zero emissions may generate net economic opportunity. Green investment can help to correct market failures such as those associated with fossil subsidies (Monasterolo and Raberto 2019), and support innovation and long-term economic growth (Stern 2015). Case studies and literature reviews find that both private financial returns (Colenbrander et al. 2016; Sudmant et al. 2016) and wider public benefits via cleaner air, reduced congestion and new employment (Dowling et al. 2022; Gouldson et al. 2018; Haines 2017; Sudmant et al. 2024), can substantially outweigh the financial costs of mitigation. Overwhelming these benefits, of course, are the value of impacts avoided from biodiversity loss (Pires et al. 2018), heat stress (Santamouris, 2020), reduced agriculture productivity (Ortiz-Bobea et al. 2021), desertification (Huang et al. 2020), and other impacts of climate change.

Accompanying the potential benefits climate action, however, are the need for reskilling millions of workers (Bowen and Kuralbayeva 2018; Robins et al. 2019), the burden on the financial system of stranded assets (Mercure et al. 2018), the need to mobilise trillions in investment capital (Giglio et al. 2021), and the challenge of unlocking social and economic systems tied to fossil fuels (Ivanova et al. 2018). In many cases these costs may be concentrated in particular geographies, or for particular subsets of the population (Green and Gambhir 2019). Challenging further the question of whether mitigation leads to net costs or benefits, different policy approaches will have different equity consequences with even small changes to policies sometimes leading to substantial changes in their socio-economic impacts (Gouldson et al. 2018; Klenert et al. 2018). And since low and zero carbon investments can generate path dependencies, especially where they are expressly designed to remake the economy (Gouldson et al. 2015; Hepburn et al. 2020), welfare and distribution effects can compound over time, magnifying and perpetuating existing inequalities.

Uncertainty around the scale and nature of both the impacts of climate change and the actions we take to address climate change make climate equity and climate justice loom even larger for policymakers. Analysis here shows a mechanism for the geographic lens of climate policymaking to lead to variation in the levels of climate action between communities, potentially magnifying variation in the welfare consequences of action and adding to this challenge. Further, the effects consistently show a skewed impact: For any climate action plan that covers less than every source of emissions, a small number of local areas will face higher than average emissions mitigation while a larger number will face lower than average emissions reductions. A relationship between deprivation and the overlap between national and local climate action plans suggests places facing the highest levels of deprivation could face the most significant impacts from data scaling.

Potential for a scaling problem, however, is not the same as the existence of a scaling problem. Variation in the sources and scales of emissions and other characteristics between local areas create the conditions for a scaling problem, but these same factors are also the reason for flexible programs of action and the devolution of responsibilities for action. In Section “Data Scaling and the Governance of Climate Action” we consider data scaling in the context of climate governance in the UK.

### Data Scaling and the Governance of Climate Action

Regional and local actors in the UK have taken different approaches to climate governance compared with the national government. The national approach to climate action, characterised by a focus on specific sources of emissions, an expanding scope of action and rising targets, is an incremental approach to climate action. The climate targets set by local authorities, of which nearly half are for 2030, by contrast, demand dramatic near-term society-wide reductions in emissions – a transformative approach to climate action (Hurlimann et al. 2021). The national approach has been driven by market-based measures and regulatory interventions while local action has to date been driven by public-private partnerships, measures to enable the climate action of non-state actors and actions to address own-source emissions (Howarth and Parsons 2021).

Even the arguments for action at each level, if not in opposition, present climate action from different perspectives. Climate action at the local and community level, often developed through place-based roadmaps (Gouldson et al. 2020) supported by programs of community engagement (Howarth and Parsons 2021), and developed with narratives that position climate change around communities, cities, homes and the countryside (Howarth and Parsons 2021), frequently make emotional appeals foundational to their case. By contrast, the national approach to climate action, with its basis in legal text and techno-economic modelling, makes logical appeals more central. A political and ideological divide thus separates local climate action in the UK and the national program of climate action.

This context is fertile ground for a scaling problem to affect climate action. Political and ideological differences stand in the way of the coordination and collaboration between actors at different scales while different approaches and narratives make the integration of climate action more challenging. Superficially it would also appear that national policy has been influenced by a scaling problem. Action to date has been focused on large sources of emissions while emissions from sectors that are smaller and that (in some cases) vary more dramatically between local areas has been lacking. The UK Climate Change Committee, for example, the independent organisation tasked with providing guidance to the UK government on climate change, has found that action on emissions from aviation, agriculture, heat and buildings has been lagging and continues to be undeveloped in existing plans (CCC 2020, 2023).

The extent, of course, that data scaling has directly affected national government policy in the UK likely to be limited. Institutional arrangements, political ideology, and the relative progress of decarbonisation technologies each likely play a much more significant role in the determination of national climate policy (Averchenkova et al. 2021; Berglund and Bailey 2023; Howarth et al. 2020; CCC 2023). Indirectly, however, analysis of data scaling contributes to our understanding of climate governance in the UK by emphasising the need for comprehensive and flexible climate action plans.

Comprehensive plans ensure the burdens and benefits that come from climate action are more evenly distributed across geographies. More comprehensive plans also support system-level interventions that are critical for the next stages of the transition (Dowling et al. 2022). Flexible climate action plans allow interventions to be adapted to local context. While analysis here shows how scaling obscures the characteristics of local areas emissions, the same process obscure social, economic, cultural and environmental features that, when embedded in the design of a policy or program, can help to make it more effective and equitable (Bulkeley and Betsill 2005). Where regional and national climate action plans fail to be comprehensive and flexible – irrespective of whether that failure emerges from data scaling or by other means—analysis here suggests the impact can be meaningful for the efficacy of climate action and the impact across local areas will be skewed: A small number of local areas will see levels of climate action that are above the average of all local areas while a larger number will see levels of climate action that are significantly below average.

## Conclusion

Several factors are understood to explain conflicting approaches to climate action—between levels of government or between actors more generally. Institutional arrangements, mobilisations of power, and ideology, for example, can all lead to conflicting climate action priorities. In the UK a significant literature critiques the Conservative government’s climate record over the last decade through the lens of these factors (Averchenkova et al. 2021; Berglund and Bailey 2023; Howarth et al. 2020). Underappreciated in existing literature, the way data is managed and analysed is shown here to be a further factor that can affect the way we understand the climate challenge.

Identifying that data scaling could affect climate action in the UK does not diminish the importance of a wider set of social, institutional and political factors for influencing the governance of climate action. Showing that the way data is managed and analysed can affect our understanding of the climate challenge, however, brings attention to methods of data analysis as an area in need of further attention. Scaling is one of a number of ways empirical methods can systematically influence our understanding of an issue and lead to unintended consequences (Simmons et al. 2011; Strube 2006). This challenge will grow if more actors become involved in the governance of climate action and as the methods we apply to determine actions become more complex and opaque.

Further research is needed to explore the potential for data scaling to affect climate action. Applying time series data and exploring other geographic contexts can add to our understanding. In addition, research considering non-state actor’s contributions to climate action and data scaling is needed. Varied levels of engagement in climate change from businesses (Revell and Blackburn 2007), widely differing levels of commitment to action (Widerberg and Pattberg 2015), and varied and sometimes conflicting approaches to action (Meath et al. 2016; Nunes et al. 2019) set the conditions for data scaling and other methodological choices to affect climate action and generate unintended consequences.
